# Antioxidant Activity in the Extracts of Two Edible Aroids

**DOI:** 10.4103/0250-474X.62242

**Published:** 2010

**Authors:** P. Mandal, T. K. Misra, I. D. Singh

**Affiliations:** Department of Botany, University of North Bengal, Darjeeling-734013, India; 1 Institute of Plantation Science and Management, University of North Bengal, Darjeeling-734 013, India

**Keywords:** *Alocasia fornicata* (Roxb.) Schott, *Alocasia macrorhiza* (Linn.) G. Don, Antioxidants, Edible aroids, Extraction, free radical scavenging

## Abstract

Two neglected species of Araceae, *Alocasia macrorhiza* (Linn.) G. Don and *Alocasia fornicata* (Roxb.) Schott are important as food and ethno medicine in Asia and Africa. Their bioefficacy is documented in the Ayurveda. The solvent extracts of different edible parts of these two species like rhizomes, leaves, roots and stolons were screened for *in vitro* antioxidant properties using standard procedures. The successive extracts in hexane, benzene, toluene, chloroform, diethyl ether, ethyl acetate and water fraction exhibited IC_50_ values in the following order, roots>rhizome>leaves for *Alocasia macrorhiza* and leaves>stolon for *Alocasia fornicate*, respectively in 2,2-diphenyl-1-picryl hydrazyl antioxidant inhibition assay. Maximum antioxidant activity was observed in diethyl ether extracts for both species. The IC_50_ values were comparable with those of quercetine and ascorbic acid as standards. These results suggest that the two aroid species have antioxidant activity in their edible parts and should be extracted using diethyl ether solvent.

The two wild aroid perennial species *A. macrorhiza* and *A. fornicata* belong to the family Araceae, are naturally grown in marshy land of tropical areas in India, Bangladesh, China, and South Africa. They are also cultivated in India and Bangladesh. Typical characteristics of *A. macrorhiza* are 4-6 ft tall in height, light green petiole, triangular shaped wide leaves and vertically geotropic rhizome with whitish roots. The characteristics of *A. fornicata* are 2-3 ft tall in height, slightly pinkish colored petiole, triangular shaped wide leaves and horizontally growing stolon. These species are commonly known as *Mana kachu* and *Shola kachu* (figs. 1 and 2) and are widely used in Ayurveda medicines in India since time immemorial.

**Fig. 1:**
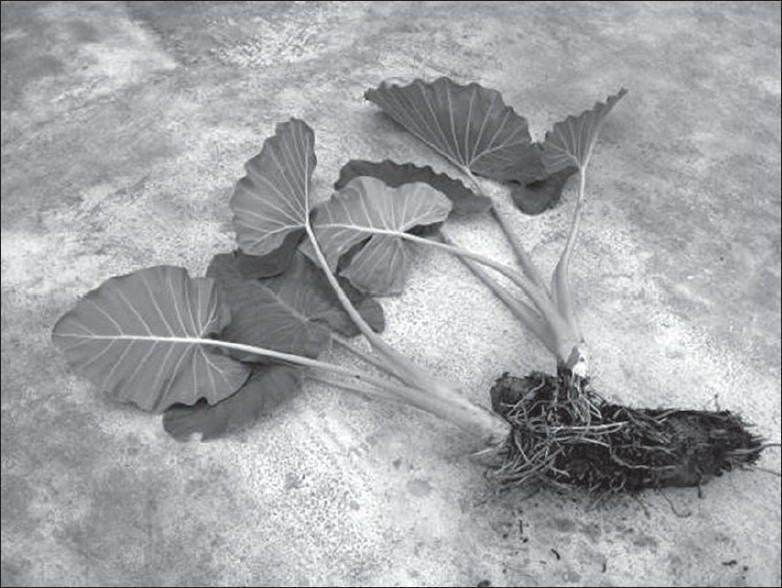
Giant taro, *Alocasia macrorhiza* (linn.) G. Don

**Fig. 2:**
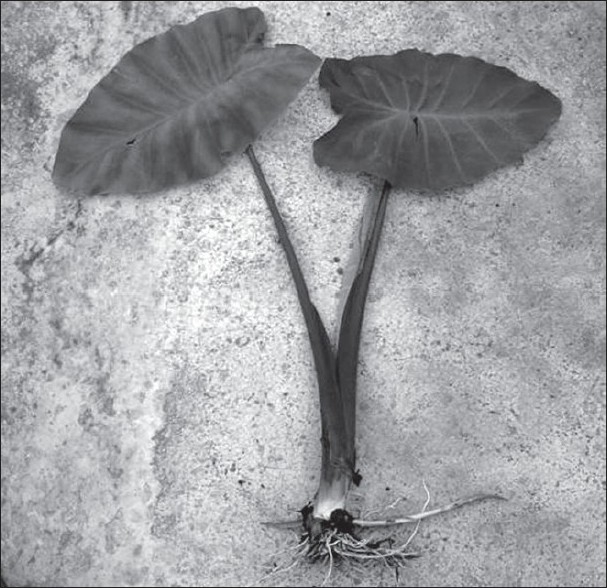
Shola kachu, *Aalocasia fornicata* (roxb.) Schott

Almost all parts of these plants are used as a food. It is also known as poor mans' food primarily due to their richness in Starch[1]. Other phyto-constituents are triglochinin and calcium oxalate[2]. The antioxidant properties of the edible parts of different plants are already established[3–5]. It is widely accepted that variety of disorders such as heart disease, chronic renal failure, diabetes, cancer, immunue dysfunction, ageing and life style related diseases are closely related to the oxidation-reduction reaction in living organisms[6–8]. Free radicals or reactive oxygen species (ROS) are formed *in vivo* from different biochemical reactions and also from the respiratory chain as a result of occasional leakage from metabolic circuits. These free radicals are the main sources of lipid peroxidation[9]. Free radicals induced oxidative stress has been implicated in the pathogenesis of wide variety of clinical disorders, resulting usually from deficiency of natural antioxidative defense[10].

There are growing industrial interests towards the production of herbal antioxidants which can be supplemented in medicine, cosmetics and nutraceuticals in modern World. This research is concerned with the demands for antioxidants in the developed World and also for those living below the poverty line in India, Africa and Bangladesh who frequently use these aroids as staple food. Therefore, a study was under taken to screen different solvent extracts of commonly used edible parts of *A. macrorhiza* and *A. fornicata* to study the *in vitro* antioxidant activities using standard procedures of DPPH based free radical scavenging activity.

The whole edible parts of *A. macrorhiza* (rhizome, roots, leaves) and *A. fornicata* (stolon and leaves) were collected from surrounding local areas of Phansideoa Block, Darjeeling, West Bengal, India in November 2006. Plant species were authenticated. The collected plant sample were cleaned, different parts were separated and dried in the shade, then powdered to 40 mesh and stored in an air tight container at room temperature. Chemicals used in the studies were 2,2-diphenyl-1-picrylhydrazyl (DPPH), hexane, benzene, toluene, chloroform, diethyl ether, ethyl acetate, quercetine, ascorbic acid and double distilled water. All chemicals used for the experiments were of analytical grade.

Different air dried ground parts (400 mg each) of these plants were soaked in 20 ml of different solvents successively [hexane, benzene, toluene, chloroform, diethyl ether, ethyl acetate and distilled water in order of increasing polarity] at room temperature over night. The extracts were filtered, and the residues were macerated for two more days with the same solvent. After filtration, the solvents were evaporated to dryness under reduced pressure at 40° and dissolved in 20 ml of methanol: water:: 4:1. Solutions of quercetine and ascorbic acid (15 μg/ml each) used as standard for *in vitro* studies were prepared in the same solvents.

The antioxidant activities of the plant extract were determined on the basis of the free radicals scavenging effect of stable DPPH free radical[11]. According to principle of the method, in the presence of an antioxidant, the purple colour intensity of DPPH solution decays and the change of absorbance are followed spectrophotometrically at 517 nm. 2 ml of DPPH was added to 200 μl of different solvent extracts solution at different concentrations. After incubation at 37° for 30 min, the absorbance of each solution was determined, the corresponding blank readings were also taken and remaining DPPH was calculated. The scavenging activity was expressed as IC_50_ (μg/ml). IC_50_ value is the concentration of sample required to scavenge 50% DPPH free radical. IC_50_ values were calculated by applying suitable regression analysis from the mean inhibitory values of DPPH radical. All analyses were done in quadruplicate.

The successive extracts of edible parts of these plant species exhibited antioxidant activity in the DPPH free radical assay as evidenced by the low IC _50_ values (Tables 1 and 2). These values were found to be less than those obtained for the reference standards; quercetine and ascorbic acid.

**TABLE 1:** ANTIOXIDANT ACTIVITY OF DIFFERENT EDIBLE PARTS OF *ALOCASIA MACRORHIZA* (LINN.) G. DON

Solvent extracts	IC_50_ values±SD* (μg/ml)		
	
	Rhizome	Leaves	Roots
Hexane	192.78±12.24	245.17±16.89	430.68±55.09
Benzene	829.12±52.66	128.07±8.82	127.72±16.34
Toluene	334.69±21.26	145.44±10.02	685.90±87.74
Chloroform	688.77±95.76	225.86±15.55	112.35±8.86
Diethyl ether	48.01± 6.68	103.39±7.12	34.51±2.72
Ethyl acetate	221.14±30.75	128.07±8.82	265.56±13.58
Aqueous	560.87±17.88	58.37±6.21	164.61±8.42

* Average of 9 determinations. Reference standard: quercetine (53.60±1.79) and ascorbic acid (78.17±4.05). All data were analyzed using SSP version 2.5 (2005).

**TABLE 2:** ANTIOXIDANT ACTIVITY OF DIFFERENT EDIBLE PARTS *ALOCASIA FORNICATA* (ROXB.) SCHOTT

Solvent extracts	IC_50_ values±SD* (μg/ml)	
	
	Leaves	Stolon
Hexane	69.35±3.50	128.73±29.49
Benzene	62.66±3.16	191.15±9.44
Toluene	400.09±20.17	180.23±41.29
Chloroform	248.85±56.14	485.22±69.15
Diethyl ether	31.11±7.02	41.23±9.44
Ethyl acetate	179.34±15.63	567.06±89.17
Aqueous	80.70±7.03	236.74±37.23

* Average of 9 determinations. Reference standard: quercetine (53.60±1.79) and ascorbic acid (78.17±4.05). All data were analyzed using SSP version 2.5 (2005).

Out of 7 extracts of *A. macrorhiza* (rhizome, roots and leaves) and two standards tested for antioxidant activity using DPPH method; the diethyl ether extract of roots and rhizome showed the maximum antioxidant activity with IC_50_ value 34.51±2.72 and 48.01±6.68 μg/ml, respectively. The diethyl ether and aqueous extracts of leaves also exhibited good quantum of antioxidant activity with lesser IC_50_ values of 103.39±7.12 and 58.37±6.21 μg/ml, respectively. However, hexane, benzene, toluene, chloroform, and ethyl acetate extracts of rhizome and roots have lower antioxidant activity with higher IC_50_ values as presented in Table 1. The known antioxidants ascorbic acid and quercetine exhibited IC_50_ value of 78.17±4.05 and 53.60±1.79μg/ml, respectively.

Diethyl ether extract of stolon and leaves of *A. fornicata* showed the maximum antioxidant activity with IC_50_ values 31.11±7.02 and 41.23±9.44 μg/ml, respectively. The other solvent extracts proved lower antioxidant activity with higher IC_50_ values than standard as presented in Table 2. Similar results were also observed during successive extraction of *Majorana syriaca*, where diethyl ether was proved to be a best solvent for extracting antioxidants[12]. But in most cases, ethyl acetate was reported as best extracting solvent for isolating antioxidant polyphenols[13].

It was confirmed from our experiments that highly hydrophobic solvents like hexane or typically polar solvents like water are unsuitable for extracting bioactive antioxidants from rhizome, roots and stolon of aroids. But in case of leaves, sufficient level of antioxidants was found in aqueous extracts which are highly comparable with reference standards. This information may be useful for preparing aroid based nutraceuticals.

This experiment has proved interestingly antioxidant activity of successive extracts of both species. They are widely used as food and Ayurvedic formulations. Certain plant species show antioxidant activity because of presence of polyphenolic constituents. Flavonoids are a broad class of lower molecular weight, secondary metabolites extraordinarily distributed in plants. Hence, the beneficial effects of *A. macrorhiza* and *A. fornicata* may be attributed to their antioxidant and chelating ability of polyphenolic constituents[14].

The present investigation has indicated the presence of good antioxidant activity in *A. macrorhiza* and *A. fornicata.* Further studies would have been required for the isolation and purification of drug to identify the active principals which may be beneficial in a number of free radical mediated disorder process. It is well known that calcium oxalate is the most important insoluble mineral found in the edible taro (*Alocasia* sp.). We have observed abundant spindle, star shaped crystals (fig. 3) of calcium oxalate, which was accumulated predominantly in the cortex tissues of roots of *A. macrorhiza*. Though existence of these types of crystals is problematic for gaining popularity but they can play effective defensive role against chewing herbivores.

**Fig. 3:**
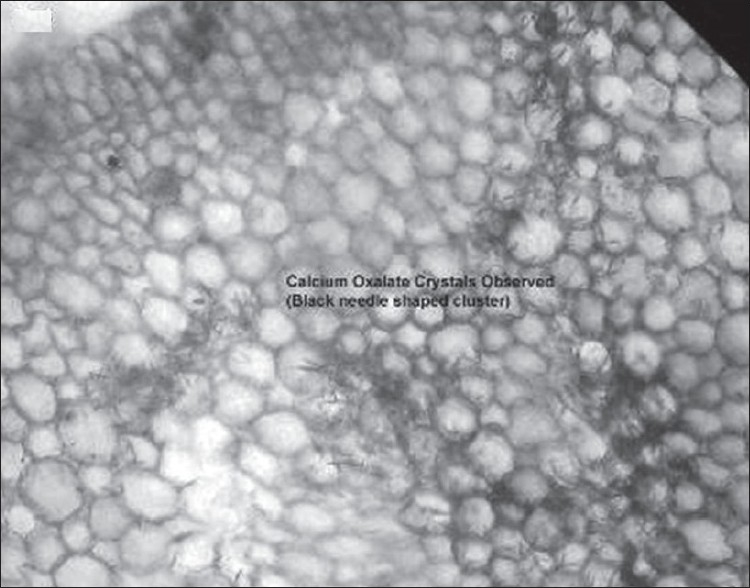
Calcium oxalate crystals in roots of giant taro *Alocasia macrorhiza* (linn.) G. Don
